# Exploring Health Care Professionals’ Perspectives on the Use of a Medication and Care Support System and Recommendations for Designing a Similar Tool for Family Caregivers: Interview Study Among Health Care Professionals

**DOI:** 10.2196/63456

**Published:** 2024-10-23

**Authors:** Aimerence Ashimwe, Nadia Davoody

**Affiliations:** 1 Karolinska Institutet Stockholm Sweden

**Keywords:** eHealth, telemedicine, mobile health, mHealth, medication management, home care, family caregivers, mobile phone

## Abstract

**Background:**

With the aging population on the rise, the demand for effective health care solutions to address adverse drug events is becoming increasingly urgent. Telemedicine has emerged as a promising solution for strengthening health care delivery in home care settings and mitigating drug errors. Due to the indispensable role of family caregivers in daily patient care, integrating digital health tools has the potential to streamline medication management processes and enhance the overall quality of patient care.

**Objective:**

This study aims to explore health care professionals’ perspectives on the use of a medication and care support system (MCSS) and collect recommendations for designing a similar tool for family caregivers.

**Methods:**

Fifteen interviews with health care professionals in a home care center were conducted. Thematic analysis was used, and 5 key themes highlighting the importance of using the MCSS tool to improve medication management in home care were identified.

**Results:**

All participants emphasized the necessity of direct communication between health care professionals and family caregivers and stated that family caregivers need comprehensive information about medication administration, patient conditions, and symptoms. Furthermore, the health care professionals recommended features and functions customized for family caregivers.

**Conclusions:**

This study underscored the importance of clear communication between health care professionals and family caregivers and the provision of comprehensive instructions to promote safe medication practices. By equipping family caregivers with essential information via a tool similar to the MCSS, a proactive approach to preventing errors and improving outcomes is advocated.

## Introduction

### Background

As the population ages, the demand for effective care solutions in home care intensifies, necessitating innovative approaches to medical care services [1]. According to studies [2,3], older adults often face complex health conditions and cognitive impairments; thus, drug therapy becomes more complex, and with an increasing number of new prescription drugs being approved and patients requiring more potent and sophisticated treatments, the likelihood of errors also increases. Studies show high rates of medication errors, with 23% to 92% in outpatient settings and nearly 50% after discharge, highlighting the need for effective interventions [4-6] Recently, the role of family caregivers in supporting older adults with daily activities, including medication management, has become vital. Recognizing this, there is a growing trend to develop digital supportive systems that lighten the burden on family caregivers while enhancing the well-being of the older adults under their care. Family caregivers often struggle with the challenges of managing multiple responsibilities, navigating complex medication regimens, and ensuring adherence to promote medication without harm, all while managing their loved ones’ cognitive conditions. In response to this pressing need, there is a call for proactive measures aimed at empowering family caregivers with tools and resources tailored to their unique needs [7-11].

In the realm of health care, eHealth refers to the integration of digital technologies and information and communications technologies into health care systems and processes. This integration aims to improve the delivery of health care services, enhance patient care, and streamline administrative tasks [12,13].

The adoption of eHealth solutions enables better coordination, data-driven decision-making, and personalized medicine, benefiting health care professionals, providers, patients, and family caregivers alike. Health care professionals gain improved access to patient data and tools for remote consultation, whereas providers streamline administrative tasks and enhance care delivery and patients, together with family caregivers, benefit from increased engagement, remote monitoring, and personalized interventions, leading to better health outcomes [1,14-16]. eHealth solutions empower patients to actively participate in their care, access educational resources, and engage in teleconsultations with health care professionals from the comfort of their homes. Moreover, digital health streamlines administrative tasks and improves care coordination and communication among multidisciplinary teams [17-20]. Building on these advancements, the role of eHealth solutions in home care has become increasingly important, particularly in countries such as Sweden, where a growing aging population receives care in their homes.

### Home Care in Sweden and the Medication and Care Support System

Home care in Sweden is a vital component of the country’s health care system, aiming to provide support and assistance to individuals who require medical or personal care in their own homes. Home care services in Sweden are organized and provided by municipal authorities, intending to promote independence, autonomy, and quality of life for individuals who may have difficulties and challenges with daily activities due to illness, disability, or aging [21,22].

Despite its well-structured and person-centered approach, several challenges persist for both patients and their family caregivers. One prominent challenge is the increasingly complex nature of medication management, particularly for older adults with multiple chronic conditions or cognitive impairments. Ensuring adherence to medication regimens, preventing medication errors, and managing potential drug interactions can pose significant hurdles. In addition, the evolving needs of individuals requiring home care demand a higher level of coordination and integration among health care providers, family caregivers, and support services, often leading to fragmented care delivery and gaps in communication [3,23,24].

**Figure 1:**
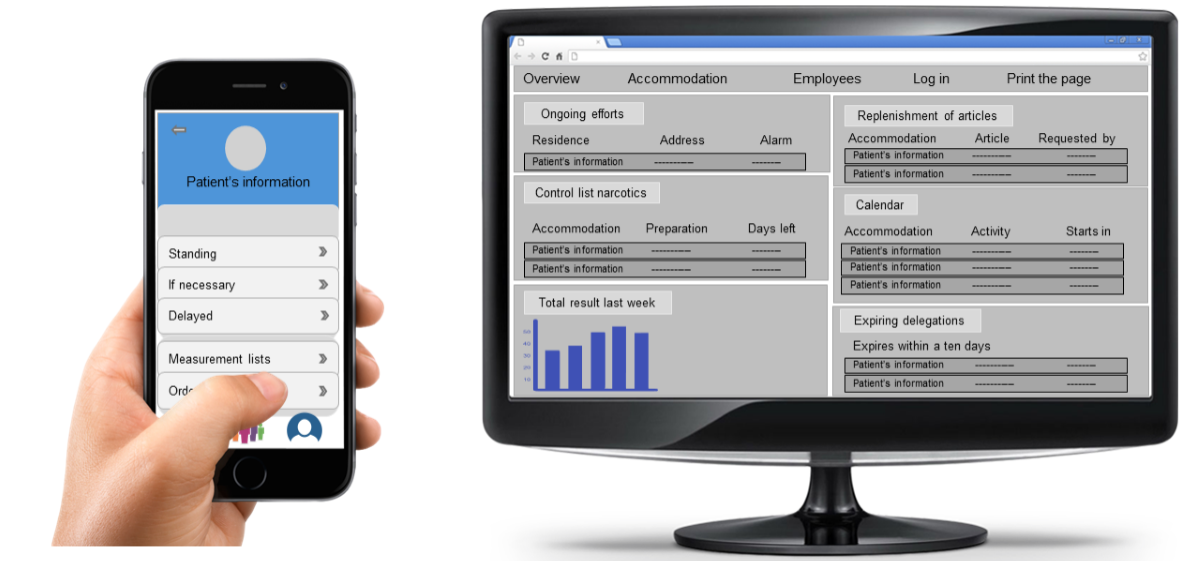
Mobile and web-based medication and care support system platform. The figure was inspired by the MCCS developed by Vitec Appva [30].

Among these challenges, the implementation of a medication management system emerges as a crucial solution to enhance the efficiency, safety, and quality of care within the home care context. By leveraging technology and tailored support mechanisms, a medication and care support system (MCSS) can assist health care professionals in the process of medication management. Moreover, a medication management system can facilitate seamless communication and collaboration among health care professionals and family caregivers, fostering a more integrated and holistic approach to care delivery [25,26]. By addressing the complexities of medication management and promoting collaboration among stakeholders, an MCSS has the potential to enhance the effectiveness and sustainability of home care services, enabling individuals to age in place with dignity and independence [27,28]. One example of a digital tool that embodies this collaborative approach to medication management is the MCSS developed by Vitec Appva [29] and widely implemented in Sweden’s home care services. The MCSS supports health care professionals in home care in medication administration, dosage tracking, and medication reconciliation, thereby reducing the risk of adverse drug events (ADEs) and promoting medication adherence. In addition to its core functions, the MCSS is extensively used by health care professionals across most home care settings in Sweden for task management, including inputting signing lists and tracking the completion of assigned duties. It is also used to simply see what the health care professionals need to do and sign off once they have completed it. It plays a crucial role in medication management in home care. With the widespread adoption of smartphones and tablets, health care professionals can access MCSS platforms from anywhere, allowing for real-time monitoring of medication schedules, reminders, administration logs, and communication tools [29]. The nurses have access to the MCSS through both a mobile app and a web platform (Figure 1 [29]). These tools allow them to customize and oversee various activities related to medication administration and patient care. With the mobile app, health care professionals can conveniently access the system from their smartphones or tablets, enabling them to stay connected and manage tasks on the go. Similarly, the web platform provides a robust interface accessible from computers or laptops, offering additional functionalities for detailed monitoring and analysis. This dual accessibility empowers nurses to tailor their approach to medication management, effectively monitor patient activities regardless of their location or device preference, assist in administrative tasks, and maintain the security of medical information [29].

However, despite the significant benefits offered by these digital solutions, challenges in medication management still persist. Studies [2,16,30,31] have shown that, even with the availability of advanced digital tools, family caregivers continue to deal with medication mismanagement in their complex caregiving responsibilities and limited communication with health care professionals. While health care professionals use the MCSS as a tool to administer medications and effectively communicate with each other, there remains a notable gap in research regarding whether a tool similar to the MCSS can also be leveraged by family caregivers to manage multiple medications, thereby mitigating medication errors in home care practices.

Studies [3,32-36] indicate that, as the population ages and drug therapy becomes more complex—with more new prescription drugs being approved and patients requiring increasingly potent and sophisticated treatments—the likelihood of errors also increases. Studies have revealed alarming statistics on medication errors and ADEs, demonstrating their profound impact on patient safety, health care providers, and health care systems. Studies [32-36] have discussed medication errors, their causes, prevention methods, and risk management strategies within the pharmacy and health care industry context. They have highlighted the importance of medication therapy as a valuable tool in treating various health conditions but also acknowledged the risks associated with its misuse. Examples of medication errors outlined in the studies include administering the wrong drug, wrong strength, or wrong dose; confusion over similar–looking or sounding drugs; incorrect routes of administration; miscalculations; and errors in prescribing and transcription. Key findings indicate that significant risk factors for medication errors include patient age of ≥60 years, inexperienced caregivers, polypharmacy (≥5 drugs), comorbidities, and multiple prescribers, which are critical to address for improving patient safety in home care [28]. In addition, a study [32] on 16,963 emergency department visits found that 3.4% were due to ADEs, with 15.1% leading to hospitalization, ADEs extending hospital stays by nearly 2 days, and medication errors adding almost 5 extra days, significantly increasing health care costs. This study emphasized the importance of implementing strategies to prevent medication errors, such as improving medication safety protocols, enhancing care delivery training, using technology for error detection and prevention, and fostering a culture of open communication and reporting within health care organizations. It underscored the need for constant caution and proactive measures to mitigate the risks associated with medication errors and ensure patient safety.

### Aim of the Study

This project aimed to investigate health care professionals’ perspectives on the use of the MCSS and gather recommendations for designing a similar tool for family caregivers. The focus was on identifying the necessary information and features essential for both health care professionals and family caregivers.

## Methods

### Study Design

In this study, an exploratory qualitative approach was adopted to investigate health care professionals’ experience of using the MCSS and recommendations for designing a similar tool for family caregivers in home care settings. To gather comprehensive insights, the researchers explored the perspectives and recommendations of health care professionals actively using the MCSS in health care delivery within home care settings [25,37,38]. The research methodology used an inductive approach, focusing on deriving insights and conclusions from collected data. Thematic analysis was conducted according to the guidelines by Braun and Clarke [38], systematically identifying recurring themes and patterns in the interview data.

### Study Setting and Participants

The interviews involved a total of 15 health care professionals and took place between February 2024 and May 2024 in Sweden. In this study, we involved health care professionals working in home care in the Nora municipality. The Nora municipality is located in the northern part of Örebro County and has approximately 10,700 inhabitants. The municipality is responsible for, among other things, home care for people who need continued health care at home. Nurses, assistant nurses, and care assistants work in home health care [39]. The study participants’ characteristics encompassed various factors, including demographic details, age distribution, tenure in home care, and experience with MCSS use. Factors such as diversity of perspectives, expertise, and experiences were considered.

The head of Nora Home Care was contacted and assisted with obtaining access to potential participants among the home care employees. Subsequently, the researchers personally met with these employees to provide a thorough explanation of the study’s purpose. Following this, interested employees who met the study’s inclusion criteria conveyed their willingness to participate via email. The participants’ eligibility was determined by their expertise in medication administration, encompassing individuals such as registered nurses, nurse assistants, care assistants, and therapists employed at Nora Home Care actively using MCSS for >2 years. These criteria helped ensure that participants possessed relevant and comprehensive experiences, perspectives, and recommendations related to the research topic while maintaining the quality and validity of the study findings [36,37,40]. The researchers sent out emails to potential participants with further details about the study objectives and attached the invitation letter along with the letter of consent. In addition, a QR code linked to a Google Form was included to facilitate scheduling for interview dates and times. Participants’ characteristics are presented in Table 1.

**Table 1:** Participant characteristics.

	Age category (y)	Occupation	Years of experience in home care	Years of MCSS^a^ use
Participant 1	45-55	Registered nurse	10-20	5-10
Participant 2	45-55	Registered nurse	30-40	5-10
Participant 3	45-55	Registered nurse	20-30	5-10
Participant 4	30-40	Occupational therapist	10-15	5-10
Participant 5	20-30	Nurse assistant	5-10	5-10
Participant 6	40-50	Nurse assistant	20-40	5-10
Participant 7	55-65	Nurse assistant	40-50	5-10
Participant 8	40-50	Nurse assistant	10-15	5-10
Participant 9	40-50	Nurse assistant	10-15	5-10
Participant 10	45-55	Nurse assistant	10-20	5-10
Participant 11	25-35	Nurse assistant	5-10	5-10
Participant 12	35-45	Nurse assistant	10-15	5-10
Participant 13	40-50	Nurse assistant	5-10	5-10
Participant 14	20-30	Carre assistant	2-5	2-5
Participant 15	35-45	Care assistant	2-5	2-5

^a^MCSS: medication and care support system.

### Data Collection

Fifteen interviews with health professionals administering medication in home care settings were conducted. Their responses centered on various aspects of medication management, including their own experiences, the role of family caregivers, the use of the MCSS tool, necessary information for caregivers, information needed by health care professionals in the MCSS from family caregivers, and recommended features for improving the tool and for designing a similar tool for family caregivers.

A pilot interview with a group of 3 nurses was conducted to assess the clarity of the research aim and interview questions. Feedback from the pilot test informed any necessary revisions. Each participant was required to sign an informed consent form before the interviews started. The interviews were recorded, and notes were taken to ensure the accuracy and comprehensiveness of the information gathered. Each interview lasted approximately 30 to 40 minutes. Given that our study included 15 participants, we closely monitored the data during collection and analysis to ensure that no new themes or insights were emerging. This point of saturation was reached when additional interviews did not produce any new or relevant information. The interviews were transcribed verbatim.

### Data Analysis

**Figure 2:**
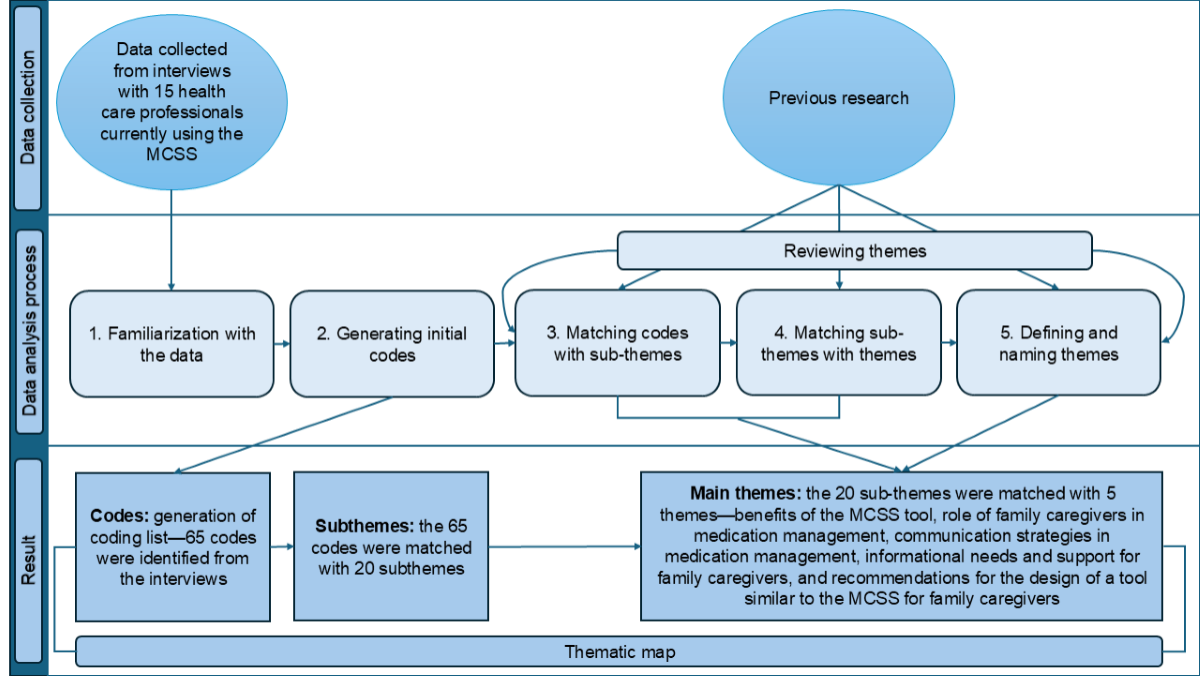
Data analysis process. MCSS: medication and care support system.

Thematic analysis was chosen as the method to analyze the data due to its inherent flexibility and systematic framework for interpreting data from qualitative research approaches. This approach facilitated the identification of recurring patterns or themes within the dataset, allowing for a nuanced exploration of the participants’ perspectives and experiences. The thematic analysis procedure, as outlined in Figure 2, inspired by the work by Matt et al [41], was guided by the step-by-step approach by Braun and Clarke [38], providing a structured methodological framework for conducting the analysis. This methodology ensured a comprehensive examination of the data, enabling the extraction of meaningful insights that contributed to addressing the research questions effectively.

The analysis began with familiarization with the data, where the first author immersed herself in the data to gain a comprehensive overview. This first step involved reading through all the transcripts of the interviews multiple times to become familiar with their content. During this phase, the author took notes, highlighted sections, and looked for recurring patterns. This step was crucial for gaining insights into the data’s context. The second step was to generate initial codes, which started by identifying specific segments that were relevant to the research question and objectives. The first author assigned initial codes to label them based on their content. The third step included matching codes with subthemes—during this step, both authors reviewed the initial codes and began to group them into clusters that reflected subthemes. A total of 20 identified subthemes represented a more specific aspect of the data compared to the broader themes. In this step, the authors examined relationships between codes and identified patterns that suggested subthemes. The fourth step was matching subthemes with themes, in which the authors combined related subthemes into broader themes that captured significant patterns in the data. The authors reviewed how subthemes fit together to form comprehensive themes that represented the major findings of the research. The last step was to describe what the themes represented and choose concise and descriptive names for the themes that accurately reflected their content. The data analysis was an iterative process involving creating new codes, subthemes, and themes based on emerging evidence.

### Ethical Considerations

This research was carried out in Sweden. According to the Swedish Ethical Review Act, the research in our submitted manuscript does not require ethics approval as it does not handle sensitive personal information (as understood by the European General Data Protection Regulation). However, ethical requirements still apply. Consequently, prospective study participants were contacted after receiving an email invitation outlining the study’s purpose. Arrangements for interviews were made via email, SMS text message, or phone call accommodating participants’ preferred timing and location. Each interview session began with a comprehensive explanation of the study’s aim, interview process, and participant rights. Before participation, all individuals provided written and verbal consent, with formal documentation signed by both the participant and the researchers. This consent form detailed study objectives, potential risks, confidentiality, and the right to withdraw. Participants were assured of anonymity and informed about how their data would be handled [42,43]. No compensation was provided to the participants in this study.

## Results

### Overview

**Figure 3:**
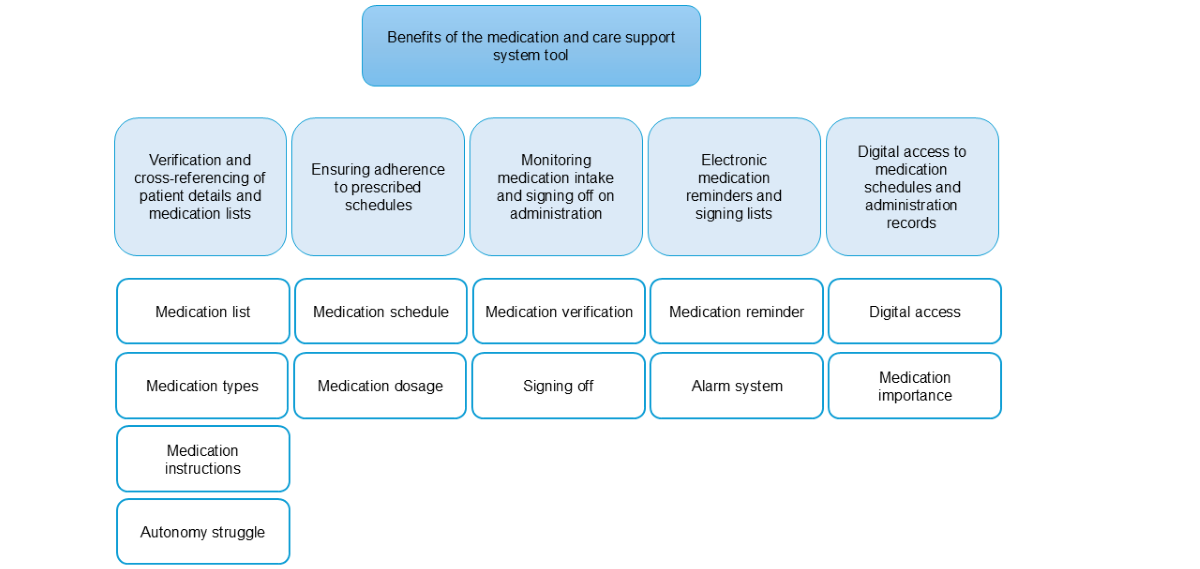
A hierarchical structure displaying the themes at the top followed by the subthemes in the middle and the corresponding codes at the bottom.

**Figure 4:**
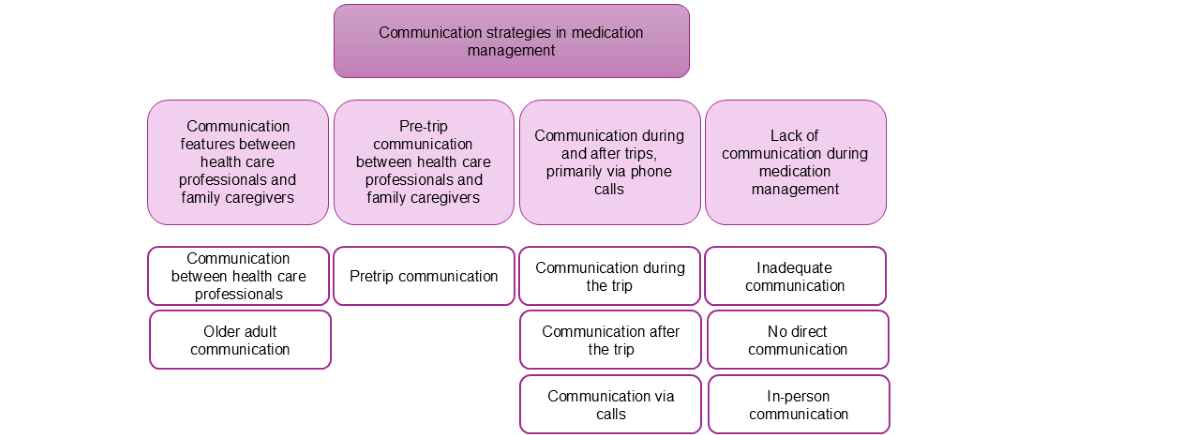
A hierarchical structure displaying the themes at the top followed by the subthemes in the middle and the corresponding codes at the bottom.

**Figure 5:**
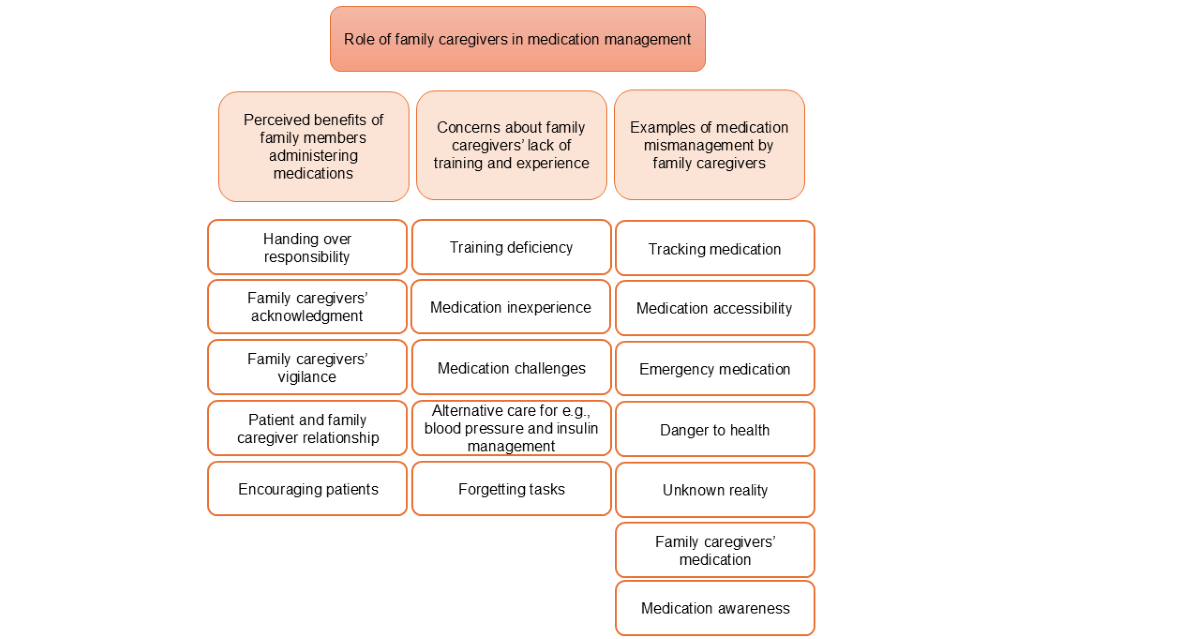
A hierarchical structure displaying the themes at the top followed by the subthemes in the middle and the corresponding codes at the bottom.

**Figure 6:**
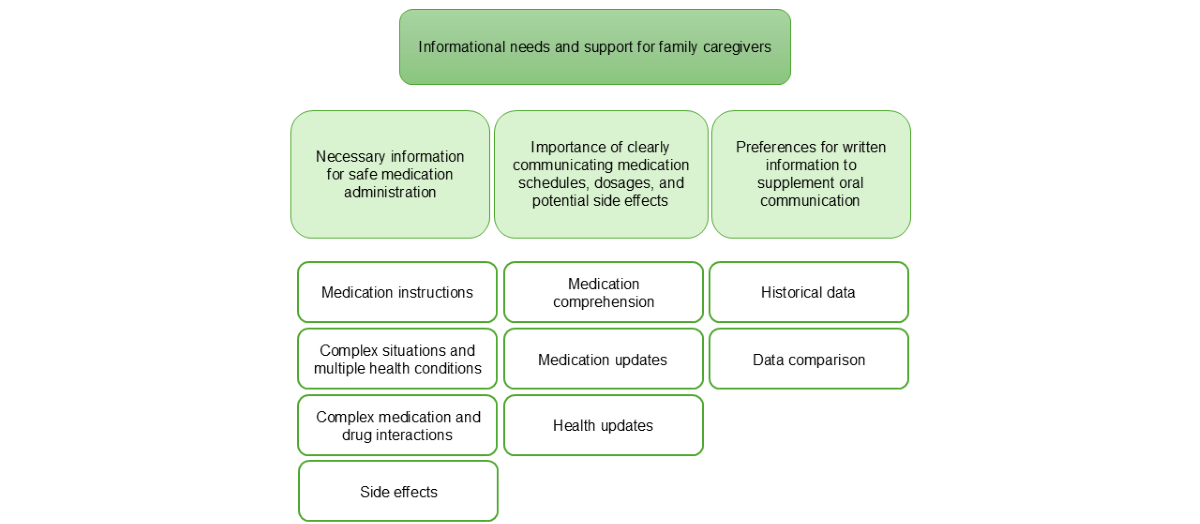
A hierarchical structure displaying the themes at the top followed by the subthemes in the middle and the corresponding codes at the bottom.

**Figure 7:**
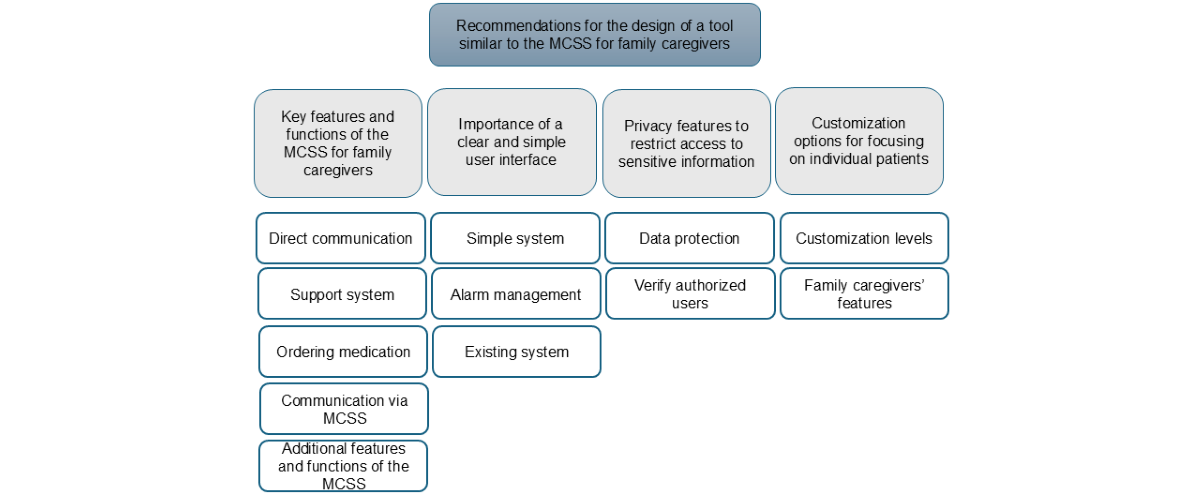
A hierarchical structure displaying the themes at the top followed by the subthemes in the middle and the corresponding codes at the bottom. MCSS: medication and care support system.

Altogether, 5 overarching themes, 20 subthemes, and 65 codes were generated (Textbox 1 and Figures 3-7). These will be showcased alongside their corresponding summarized meaning units, with participants identified by their designated number following their quotations.

**An overview of the themes and subthemes.**

**Benefits of the medication and care support system tool**

- Verification and cross-referencing of patient details and medication lists
- Ensuring adherence to prescribed schedules
- Monitoring medication intake and signing off on administration
- Electronic medication reminders and signing lists
- Digital access to medication schedules and administration records

**Role of family caregivers in medication management**

- Perceived benefits of family members administering medications
- Concerns about family caregivers’ lack of training and experience
- Examples of medication mismanagement by family caregivers

**Communication strategies in medication management**

- Communication features between health care professionals and family caregivers
- Pretrip communication between health care professionals and family caregivers
- Communication during and after trips, primarily via phone calls
- Lack of direct communication during medication management periods

**Informational needs and support for family caregivers**

- Necessary information for safe medication administration
- Importance of clearly communicating medication schedules, dosages, and potential side effects
- Preferences for written information to supplement oral communication

**Recommendations for the design of a tool similar to the MCSS for family caregivers**

- Key features and functions of a similar MCSS for family caregivers
- Importance of a clear and simple user interface
- Privacy features to restrict access to sensitive information
- Customization options for focusing on individual patients

### Theme 1: Benefits of the MCSS Tool

#### Overview

A hierarchical structure displaying the themes at the top followed by the subthemes in the middle and the corresponding codes at the bottom (Figure 3). The participants mentioned that the MCSS tool presents a comprehensive solution designed to streamline the medication administration process. They revealed that its functionality lies in the abilities outlined in the following sections.

#### Verification and Cross-Referencing of Patient Details and Medication Lists

The MCSS tool helps health care professionals verify patients’ details on the app and cross-reference them with medication lists at the patients’ home to ensure accuracy and consistency in medication administration:

We use MCCS in the municipality to ensure and know that the care recipients have received their prescribed medication. It is a way to verify that it has been done or check exactly as prescribed in the system.

Participant 1

When I arrive at the patient’s [home], I open the app and go to the person who is supposed to receive medication. I verify that the name and person match, and I check the prescription list they have in their binder at home, cross-referencing it with what is displayed in the Appva MCSS app.

Participant 6

#### Ensuring Adherence to Prescribed Schedules

The participants expressed that the MCSS takes into account any specific instructions for medication timing and dosage to maintain adherence to the treatment plan. Participants discussed that this feature provides a way to track and manage medication schedules effectively for different household members. Similarly, by selecting the correct person within the app, one can view details about prescribed medications, including which medicines should be administered, the dosage instructions, and the specific times when each medication should be taken:

You enter the app under the right person. Then you can also check the prescription action that is available at everyone’s home. It states which medicine to give, how to give it, and at what time.

Participant 5

The participants also described how occupational therapists and physiotherapists use the MCSS to organize training sessions and provide feedback. They mentioned that, during home training visits, the occupational therapists collaborate with physiotherapists to address patients’ training needs:

The process, it’s about when we’re at home for training visits. We can say that physiotherapists are meeting someone who has a training need where staff need to be present. Then we go through what type of training program [they have], then we put in the MCSS description of the training and how often, how long approximately during the day, and then we provide feedback to both patients and staff.

Participant 4

#### Monitoring Medication Intake and Signing off on Administration

The participants discussed the MCSS’s ability to monitor medication intake, and they sign off on administration on the app to confirm that the medication has been given as prescribed:

Once I have retrieved the medication, I observe that the person has taken or swallowed it. If I have administered the medication myself, I then sign off to confirm that it is complete.

Participant 6

#### Electronic Medication Reminders and Signing Lists

The participants appreciated the implementation of electronic medication reminders within the system that prompt health care professionals about scheduled doses. They also highlighted the importance of electronic signing lists through the MCSS to track medication administration accurately and discussed that this system promotes self-awareness by flagging instances in which medication is not taken or documented properly, ensuring optimal care:

An alert may come up. I can see that the patient or care recipient may not have taken their medication. Perhaps they have taken it, but it’s not signed for. This is to ensure good self-awareness in medication management...

Participant 1

#### Digital Access to Medication Schedules and Administration Records

The participants emphasized the value of digital access to medication schedules and administration records, enabling health care professionals to have comprehensive and up-to-date information about medication management:

Is a digital platform that facilitates the management of medication and care for our patients. Through MCSS, we can schedule medication, keep track of dosages, and improve communication among healthcare professionals. Within the system, you can see whether medications have been administered or not.

Participant 10

Following the exploration of the benefits provided by the MCSS tool, it is equally important to examine the crucial role that family caregivers play in medication management. This next section delves into the perceived benefits and challenges faced by family caregivers, highlighting their experiences and the potential risks associated with their involvement in administering medications.

### Theme 2: Role of Family Caregivers in Medication Management

#### Overview

A hierarchical structure displaying the themes at the top followed by the subthemes in the middle and the corresponding codes at the bottom (Figure 4). The participants described how family members may step in to take on tasks typically performed by health care professionals and acknowledged the significant role of family members in the care of the patients. However, the participants exposed some challenges that arose when family caregivers had the responsibility of administering medication.

#### Perceived Benefits of Family Members Administering Medications

Participants recognized the value of family members assisting with medication administration and acknowledged that this involvement fosters a sense of collaboration and shared responsibility in the patient’s care:

In general, it is considered good that relatives take responsibility for administering medications. It allows the patient to have autonomy and independence. That one is not so dependent on the assistance of healthcare personnel.

Participant 1

Actually, relatives take over what the staff usually do when they take care of the patient themselves. It can be temporary, for example, I am visiting my mom now at lunch and I will stay until 3 o’clock so I will give her medication at 2 o’clock.

Participant 2

#### Concerns About Family Caregivers’ Lack of Training and Experience

Despite the benefits, the participants expressed concerns about the potential risks associated with family members administering medications. They noted the lack of formal training and expertise among family caregivers, which could lead to errors or omissions in medication administration:

Yes, it’s quite a big responsibility that family members take on. It’s important that they receive enough information, so they know which medications to give and at what times. There may also be a medication that shouldn’t be administered if the person’s condition is worse or changes in a certain way. It’s quite a significant responsibility to assume...It actually carries the same risks as healthcare professionals. But the risk becomes greater because family members are inexperienced and cannot read medication lists. They don’t have the training that healthcare professionals have.

Participant 3

#### Examples of Medication Mismanagement by Family Caregivers

Participants shared examples in which medication mismanagement occurred when family caregivers were responsible for administration. These examples included cases in which medications were not taken as prescribed or doses were missed altogether. They also speculated that the family caregivers may not have fully understood the patient’s cognitive state or failed to double-check medication adherence. They also noted that those incidents highlighted the importance and need for clear communication and caution in medication management even when delegated to family members:

There was one incident that comes to mind where a woman we sent home with her medication, as her relatives had informed us, she would be going home to her daughter and taking her medication with her. But when she returned home after about a week, she hadn’t taken any of her medication. She had medication three times a day...In this situation, I think that perhaps the relatives hadn’t realized that the person wasn’t entirely clear in her thinking. Or maybe they didn’t double-check properly...but in this case, the person hadn’t taken their medication. So, she brought half of the pill dispenser back home.

Participant 6

It has happened to me actually. I sent medication to a patient with dementia. And the family was supposed to take responsibility for the medication. We sent the medication box that he should take, and it was only medication for two days, but the patient took all the medication. We don’t know if he took all the medications or if he threw them away...This is a bit of a problem if the family members know nothing about the medication. There are always problems with family members and patients when they are going to leave or visit.

Participant 9

Building on the discussion of family caregivers’ roles in medication management, it is essential to consider how communication strategies impact this process. The following section explores the various communication features and challenges between health care professionals and family caregivers, focusing on the critical moments before, during, and after trips and the implications of inadequate direct communication during the medication management period.

### Theme 3: Communication Strategies in Medication Management

#### Overview

A hierarchical structure displaying the themes at the top followed by the subthemes in the middle and the corresponding codes at the bottom (Figure 5). The participants described communication strategies used between health care professionals and family caregivers typically occurring before, during, and after trips, primarily in person or through phone calls. However, the participants mentioned that they had been contacted only before the trips but never been contacted while the patient was away, whereas both family caregivers and care recipients are encouraged to communicate with health care professionals whenever they have questions if they find any changes or seek advice regarding medication management.

#### Communication Features Between Health Care Professionals and Family Caregivers

The participants discussed the situation regarding communication between health care professionals and family caregivers:

Currently, we communicate with family members regarding medication administration through phone calls and in-person meetings before they travel with the patient.

Participant 11

#### Pretrip Communication Between Health Care Professionals and Family Caregivers

The participants discussed the importance of communication between health care professionals and caregivers before a trip. They mentioned that, typically, communication occurs before the trip, where caregivers inform health care professionals about their travel plans and inquire about managing medications during their absence:

Yes, we plan together if someone is going away for two weeks, we say, so then it’s the nurse who is informed and dispenses medications for the entire period.

Participant 3

#### Communication During and After Trips, Primarily via Phone Calls

The participants highlighted a lack of ongoing communication during and after trips. They expressed that, during trips, there was no communication with family caregivers and the same happened afterward except for health care professionals possibly asking how it went. In addition, participants explained that, after trips, communication remained minimal and mentioned that this approach may not provide timely updates and not ensure that everyone involved is informed about the medication regimen while many changes may occur during the absence of the patient:

But during and after, we don’t have any direct communication in that way. Instead, the entire responsibility is left, and it’s explained beforehand. During that time, we have no communication with them, and not afterward either. Except that one can ask how it went.

Participant 5

#### Lack of Direct Communication During Medication Management Periods

The participants acknowledged challenges related to trusting family caregivers to manage medications correctly. They mentioned the difficulty in verifying whether medications were taken properly, especially when the responsibility is entirely on the family caregiver because they cannot sign off on the medication:

It’s a bit tricky for us as healthcare professionals; we don’t receive any information about the patient, whether they have taken medication at the right time, or they haven’t taken all their medications.

Participant 9

Often challenges related to them not having enough medication support. Since we are not with them, we don’t know how it works for them.

Participant 10

The participants continued to discuss the importance of planning travels well in advance to ensure smooth coordination; otherwise, it resulted in patients not having enough medication before the trip. They mentioned the challenges regarding the fact that, in such cases, arrangements need to be made for the patient to receive the medication dispenser at the pharmacy where their family caregiver lives:

Also, another challenge is that when family members want to take care of the medications, they usually order upcoming medication not in good time, they always say it at the last minute, and it usually takes time to fix. It can affect the patient’s illness, for example, when they are not close.

Participant 11

Effective communication is crucial, but ensuring that family caregivers have the right information is equally important. The next section delves into the informational needs of family caregivers, emphasizing the significance of clear instructions and understanding medication schedules and potential side effects and the preference for written materials to support oral communication.

### Theme 4: Informational Needs and Support for Family Caregivers

#### Overview

A hierarchical structure displaying the themes at the top followed by the subthemes in the middle and the corresponding codes at the bottom (Figure 6). The participants highlighted the need for detailed information about medication administration. They stressed the importance of providing written instructions, especially for complex situations, to ensure that family members can effectively assist with medication management.

#### Necessary Information for Safe Medication Administration

The participants emphasized the importance of having updated information regarding any changes or updates to medication prescriptions to ensure that family caregivers and health care professionals administer the correct medications according to the latest prescriptions:

They can see there, for example, at nine o’clock it should be this APO on the bag, and then at twelve, it should be insulin and APO, like that.

Participant 5

They should know why they’re taking the medication, which medication it is, and what illness it’s for.

Participant 12

Participants highlighted the importance of family caregivers being updated on any changes in the medication list, such as medication names or any changes in patients’ conditions that needed to be reported to health care professionals through the MCSS. They expressed that this information is crucial to health care practices to ensure that everyone is informed and involved in the patient’s care:

Yes, one thing that can be difficult is when it says that they should give a certain medication with certain names, and then a similar product with a different name has come from the pharmacy. Then it’s hard to know what it is I should give... You have to update them if there have been any changes. I always try to inform them when I can. When the patient themselves doesn’t have such a good memory or understanding of their health condition, then I usually contact relatives and inform them.

Participant 2

#### Importance of Clearly Communicating Medication Schedules, Dosages, and Potential Side Effects

The participants stressed that clear communication of medication schedules and dosages is crucial for safe administration and that the information does not need to be too much to read. They expressed that family caregivers and patients need to understand only when and how to take each medication to ensure adherence to the prescribed regimen:

To have information about medication, regimens, dosages, potential side effects, and what to do in case of medical emergencies. Communicating these details clearly and regularly is important to ensure safe and effective care.

Participant 10

They also emphasized that communicating potential side effects of medications is essential for family caregivers and patients should be aware of possible adverse reactions so that they can monitor them and seek medical attention if necessary:

When the patient is about to feel nauseous and vomit, maybe you understand what to do then with the medications. And sometimes that type of information needs to be written down as well, so it can be given to relatives, not just verbally; it can be a lot of information at once.

Participant 3

The family members have to be informed about what can sometimes happen to patients after taking such medication.

Participant 9

#### Preferences for Written Information to Supplement Oral Communication

Participants highlighted the written instructions on how to administer each medication, including dosage, route, and any special instructions or preparations that are necessary to avoid errors and make good decisions in administration:

I recommend that all information exchanged between healthcare professionals, relatives, and patients should be in written form rather than on the phone, as it can cause misunderstandings...Relatives receive the same information about medications as the patient does, including the illness...

Participant 13

Building on the importance of clear communication and information, it is essential to consider how technology can support family caregivers in managing medication. The following section explores recommendations for the design of a tool similar to the MCSS, highlighting the need for key features such as a user-friendly interface, strong privacy protections, and customizable options to meet the unique needs of individual patients.

### Theme 5: Recommendations for the Design of a Tool Similar to the MCSS for Family Caregivers

A hierarchical structure displaying the themes at the top followed by the subthemes in the middle and the corresponding codes at the bottom (Figure 7). The participants recommended the design of a system similar to the MCSS for the managing of medication and health care information by family caregivers.

#### Key Features and Functions of a Similar MCSS for Family Caregivers

On the basis of the existing MCSS, participants highlighted specific features and their roles that can help family caregivers effectively manage medication.

#### Alarm System or Medication Reminder

Participants emphasized the importance of a reminder function within the system to prevent missed doses or overdoses. They suggested that reminders could be sent via SMS text messages or notifications to ensure timely medication administration:

I’m thinking that perhaps one could implement receiving a text message or something to a relative’s phone when it’s time for medication.

Participant 6

Electronic medication reminders are important, digital signing lists that show if medications have been administered; this gives relatives the opportunity to communicate directly with healthcare staff...These features help to increase the efficiency and safety of medication administration, as well as promote collaboration and communication between healthcare professionals and relatives.

Participant 10

#### Medication List (Dosage and Schedule)

Participants highlighted the need for easy access to a comprehensive list of medications, including both regular and as-needed (nonregular) medications. They suggested that the system should provide clear instructions regarding dosage, timing, and potential interactions to assist family caregivers in managing medications effectively:

If family caregivers can go in and get an overview when the care recipient has medication that you want to sign for. So, I think MCSS would help there...It’s about seeing the times when medication should be given. If it’s every hour or an hour before it’s completely okay...Family caregivers should log in and check for as-needed medications, to see when and how often they can be given...

Participant 1

I’m thinking that as we have with MCSS, they can see there, for example, at nine o’clock it should be this APO in the bag, and then at twelve it should be insulin and APO like that.

Participant 5

#### As-Needed Medication Access

The discussion focused on the importance of medication access and patient safety. However, participants described a situation in which a family member is responsible for administering scheduled medication to a patient but access to additional medication (as needed) is restricted. If the patient suddenly needed medication during this time, the family member might not be able to provide it, leading to frustration or danger to patients:

...When a relative who is with a patient has received both a pharmacy bag and doses from the healthcare staff to be given at the next scheduled time, which the relatives will be responsible for and the rest is locked up in a cabinet at the patient’s home and then that period begins when the patient feels unwell and needs medication, and then they don’t have access to them, such as as-needed medication. So, it can be frustrating for them...In the worst cases, we lock up medications to have better control.

Participant 2

Participants discussed the crucial importance of medication management, particularly concerning narcotics, in home patient care. They noted that these medications are securely stored and only accessible to health care professionals due to their high vigilance requirement to avoid the potential risks associated with narcotics. This limited access can pose challenges during emergencies when family caregivers are responsible for medication administration.

#### Medication Instructions

Participants suggested that the system should provide detailed information about each medication, including its purpose, administration instructions, and potential side effects. They emphasized the importance of clear communication to reassure family caregivers and ensure safe medication administration:

Yes, perhaps relatives would need to have a description of what the medication is for in MCSS because it provides a sense of security to know what I am putting into my mouth, so that there can also be some help text provided not to give in these symptoms, for example. Or consider always giving with food or not with food and such things...Or if there’s a particular pill that is important to give at an exact time, one could have extra instruction for such matters, because we don’t have that now, as the staff can manage it when it’s not needed in the same way now. But for relatives, it would be very good; it would reassure them in that case; it would be a completely new function.

Participant 3

#### Signing off and Medication Confirmation

Participants discussed the importance of a feature that allows family caregivers to confirm medication administration. This confirmation could serve as a record for monitoring adherence and tracking progress, providing reassurance for both caregivers and health care professionals:

I believe that signing list for training and rehabilitation is the best feature of relatives. Then it’s also the case that we have used it, sometimes I have probably used it just to get a confirmation that it’s carried out. It becomes a reassurance for us when we delegate training or rehabilitation, that we see that it’s being done in the way we’ve agreed upon with patients and relatives.

Participant 4

And that it becomes clear for them too that they could simultaneously sign so that we still get an overview that their medication has been given at the same time so that they can also check themselves that the medication has indeed been given and that they have signed that they have given it.

Participant 5

#### Preordering Medication

Participants suggested that the family caregiver’s system should include a feature for preordering medications, particularly for travel preparation. This would ensure that family caregivers have an adequate supply of medications on hand even when they are away from home:

Yes, the other functions, like ordering medication, because when they are not around, they have to have enough medication otherwise it’s difficult to deliver medication where they are.

Participant 2

#### Importance of a Clear and Simple User Interface

Participants advocated for the importance of a system that is easy to navigate, particularly for individuals lacking technical expertise, older family caregivers, or busy working individuals. They suggested that it should display only essential information to avoid overwhelming users:

It shouldn’t be anything complicated, it’s just that the time and date are written. It doesn’t need to be anything difficult...Sometimes they may think it’s too much information; it should be a bit simple. If it’s just a medication bag, maybe there should just be an option to press or sign, or if you’re giving insulin. That it’s something simple. It can also be older people who find such things difficult.

Participant 7

And it would be a system that is easier for them because some don’t have any technical training.

Participant 11

#### Privacy Features to Restrict Access to Sensitive Information

The participants expressed the importance of ensuring privacy, security, and personalized access to health care information through the system. They emphasized that the system should prevent unauthorized access; only authorized individuals such as close relatives should have access to the system and specific patient data:

The functions to hide are the personal identification number, or so that someone else cannot log in...It’s just that only closest relatives receive all the information, for example, the personal identification number and what medication it is.

Participant 8

#### Customization Options for Focusing on Individual Patients

The participants stressed the significance of customization options for focusing on specific patients, particularly for family caregivers. They highlighted the importance of ensuring that family caregivers only have access to information relevant to their particular patient rather than being able to view data for all patients, as health care professionals can:

Then the system must be installed on their phones. And then they should only have access to that patient and no one else.

Participant 2

## Discussion

### Principal Findings and Comparison With Prior Work

This study delved into 15 health care professionals’ perspectives regarding medication management for older adults receiving home care, with a specific focus on exploring the current digital support tool being used (MCSS). This study aimed to explore health care professionals’ perspectives and gather recommendations regarding the design of a similar tool for family caregivers. The findings identified 5 key themes: benefits of the MCSS, role of family caregivers in medication management, communication strategies in medication management, informational needs and support for family caregivers, and recommendations for the design of a tool similar to the MCSS for family caregivers.

Similarly, this study found 10 key MCSS features—alarm system or medication reminder, medication list, medication dosage and schedule, as-needed medication access, medication instructions, signing off and medication confirmation, preordering medication, user-friendly interfaces, privacy features, and customization options—that are crucial for family caregivers to manage medication and have direct communication with health care professionals.

The essential features of the MCSS not only support effective medication management for family caregivers but also reflect the broader impact of the system, as highlighted by the findings, which confirm the MCSS’s role in simplifying medication administration processes and facilitating direct communication with health care professionals. All participants expressed that the MCSS offers the benefits of verifying patient details, ensuring adherence to prescribed schedules, and providing electronic reminders and direct communication with health care professionals. In addition to the benefits of the MCSS tool, the crucial role of family caregivers, also known as informal caregivers, in providing unpaid support to patients cannot be overlooked. Studies [16,19,44] have shown that family caregivers offer a wide array of unpaid assistance. On the basis of these studies, family caregivers play a crucial role in medication management, particularly as health care systems increasingly rely on them to support patient care at home. These studies emphasize the growing responsibilities of family caregivers, especially in palliative care settings, where they are often tasked with managing complex medication regimens. These studies [14,19,44] highlight the fact that family caregivers are often responsible for ensuring that medication is administered correctly, managing complex medication schedules, and addressing any side effects or interactions. However, these responsibilities can be overwhelming, especially when caregivers lack formal training or adequate support. These studies highlight the need for robust support systems to ensure that family caregivers effectively handle these responsibilities. In our study, participants acknowledged the significant responsibility shouldered by family caregivers in administering medications and the need for a support system. In addition, our study highlighted insufficient communication between health care professionals and family caregivers. As the primary communication occurred before handing over medication responsibility to family caregivers during trips with patients, participants noted challenges in maintaining communication during and after the trips. The MCSS can help ease these challenges by providing tools that streamline medication management, offer clear instructions, and facilitate ongoing communication with health care professionals.

Beyond communication challenges, participants also expressed concerns regarding the lack of training and experience among family caregivers in medication administration, which led to instances of medication mismanagement. This underscored the importance of addressing educational gaps by providing an adequate support system to family caregivers to ensure safe medication practices, as was mentioned in previous studies [28,44,45]. To address this challenge identified both in these studies and in our study, the MCSS should include comprehensive training modules that help family caregivers understand how to use the system effectively. These modules could be delivered through the app or via web-based tutorials, ensuring that family caregivers feel confident in managing medications.

Moreover, challenges related to verifying medication administration under family caregiver responsibility were expressed. The participants highlighted the importance of the MCSS, which is built with the ability to ensure proper medication administration and provide electronic reminders. They discussed that the system provides detailed medication information and sends reminders when it is time to administer medication or when time passes. In addition, previous studies have described the importance of a digital system that provides comprehensive written instructions [28,29,45] for safe medication administration and clear communication regarding medication schedules, dosages, and potential side effects. The findings of these studies align closely with our study’s findings regarding the role and support needs of family caregivers and the importance of support tools that provide comprehensive and accessible information for medication management. Using a digital system among family caregivers was suggested as a crucial approach to promoting proper medication adherence and reducing the likelihood of errors associated with ADEs. These results are aligned with the results of previous studies that highlight the crucial role of digital tools in reducing medication errors and improving patient care. These studies demonstrate that telehealth interventions and telemedicine can enhance caregiver support and reduce medication management errors [10,17,20,28,46,47]. These studies emphasize the integration of telehealth for monitoring chronic conditions, reinforcing the need for a comprehensive system that supports caregivers in managing complex medication regimes. In addition, these studies discuss the prevalence of medication errors and adverse events, which our study aligns with by advocating for enhanced MCSS features to mitigate these issues.

Building on this, the participants highlighted how specific features, such as confirmation processes, could address the challenges identified in previous studies and further enhance the MCSS’s effectiveness in managing medication errors and adverse events.

Our study identified that the design of an MCSS similar to that used by health care professionals is necessary for family caregivers to bridge the communication gap and improve medication management. However, while our study explored health care professionals’ perspectives on this issue, more studies are needed to investigate this subject from family caregivers’ and patients’ perspectives. In addition, the design recommendations should be verified based on family caregivers’ and patients’ input.

### Limitations of the Study

Some of the limitations of this study include the small sample of participants. While the in-depth nature of the interviews provided rich insights, the limited sample size may restrict the generalizability of the findings to broader populations of health care professionals working in diverse home care contexts. Future research should aim to increase the sample size to enhance the representativeness of the findings, strengthen the generalizability of the results, and ensure the broader applicability of the findings. Another notable weakness is the lack of inclusion of patient and family caregivers’ perspectives in the study. By primarily focusing on health care professionals’ viewpoints, this research overlooks the experiences and preferences of older adults receiving home care and of their family caregivers. By engaging patients and family caregivers in future studies, their perspectives and needs can be considered. Future research should prioritize incorporating the viewpoints of both patients and their family caregivers and the development, implementation, and evaluation of such interventions to assess their effectiveness and feasibility in real-world contexts.

### Conclusions

This study delved into the complexities of medication management among older adults in home care, focusing on health care professionals’ perspectives and the use of the MCSS. Through interviews, this study uncovered critical themes and recommendations, emphasizing the need to address gaps in medication management practices, particularly concerning similar support tools for family caregivers. In conclusion, incorporating the recommended design features, such as customizable interfaces and enhanced privacy controls, into the MCSS can improve usability and effectiveness for family caregivers. These enhancements are expected to facilitate better medication management, reduce errors, and strengthen communication with health care professionals. By equipping family caregivers with essential information via a tool similar to the MCSS, a proactive approach to preventing errors and improving outcomes is advocated. However, the impact of these improvements should be verified in future studies to confirm their effectiveness and address any emerging challenges.

## Abbreviations

ADE: adverse drug event
MCSS: medication and care support system
